# Sulfur quantum dot-based “ON–OFF–ON” fluorescence platform for detection and bioimaging of Cr(vi) and ascorbic acid in complex environmental matrices and biological tissues†

**DOI:** 10.1039/d1ra00401h

**Published:** 2021-03-11

**Authors:** Mengke Xia, He Mei, Qiuhui Qian, Randy A. Dahlgren, Ming Gao, Xuedong Wang

**Affiliations:** School of Environmental Science and Engineering, Suzhou University of Science and Technology Suzhou 215009 China zjuwxd@163.com gaoming@usts.edu.cn +86 512 6809 5950 +86 512 6809 5950; College of Public Health and Management, Wenzhou Medical University Wenzhou 325035 China

## Abstract

Based on an “assembling–fission” principle, stable sulfur quantum dots (SQDs) were synthesized using sublimed sulfur as a precursor and PEG-400 as a passivator. The fluorescence intensities (FIs) of SQDs were efficiently quenched by Cr(vi) due to formation of SQD/Cr(vi) complexes through the inner-filter effect. When ascorbic acid (AA) was introduced into the SQD/Cr(vi) system, SQD fluorescence was restored due to AA-induced reduction of Cr(vi) to Cr(iii). Consequently, a SQD-based “ON–OFF–ON” platform was constructed for sequential detection of Cr(vi) and AA. Under optimized conditions, the FIs of SQDs were linearly dependent on the concentrations of Cr(vi) and AA, yielding linear ranges of 0.005–1.5 and 0.01–5.5 mM with detection limits of 1.5 and 3 μM, respectively, in waters, serum and tablet samples. After a 24 h incubation, the SQDs displayed strong, quenched and recovered blue fluorescence, respectively, in the SQD, SQD/AAO/Cr(vi) and SQD/Cr(vi) systems in live HeLa cells and zebrafish embryos/larvae. A blue fluorescence was displayed in the yolk of zebrafish embryos, and yolk and head of larvae. This study demonstrates the efficacy of SQD systems for environmental and biological applications in complex matrices, and for direct observation of Cr bioaccumulation in organisms by bioimaging.

## Abstract 2

A SQDs-based “ON–OFF–ON” platform is constructed for detection of Cr(vi) and AA. SQDs are feasible for bioimaging of zebrafish embryos/larvae and HeLa cells.

## Graphical abstract

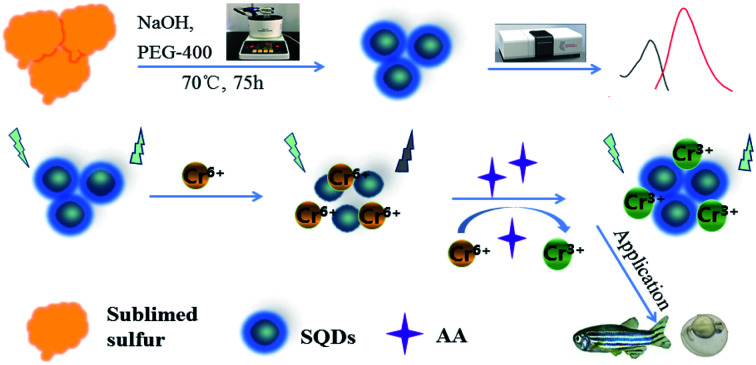

## 1. Introduction

Luminescent quantum dots (QDs) are widely used in the fields of optoelectronic devices, biomarkers and biomedicine due to their unique physicochemical properties.^1,2^ QDs with outstanding performance include nanoprobes/nanosensors such as CdSe, CdSe/ZnS, InP/ZnS and CuInS_2_/ZnS.^3,4^ However, these metal-based QDs pose a human health concern and their preparation cost is relatively high. Consequently, development of novel QDs that produce light at low cost and possess low toxicity is highly desirable. In the last decade, research focused on developing heavy-metal-free QDs, especially pure elemental formulations, such as phosphorus, carbon, sulfur and silicon QDs, which have the desirable properties of low toxicity, good dispersibility, stable photoluminescence and so on.^5,6^

Compared to carbon and phosphorus-based QDs, sulfur quantum dots (SQDs) are often regarded as a promising next-generation nanomaterial because of their advantageous properties, such as excellent aqueous dispersibility, photostability, environmental friendliness and inherent antibacterial properties.^7–10^ SQDs (<10 nm) are mainly composed of elemental sulfur nanoparticles and surface-modified groups. Li *et al.* (2014) synthesized SQDs using a three-step phase interface reaction, but achieved a fluorescence quantum yield of only 0.5%.^7^ A herbal compound was used by Kouzegaran *et al.* (2017) as a surfactant to catalyze the precipitation reaction of sodium thiosulfate solution to enhance the synthesis of SQDs.^11^ To address low quantum yield, Shen and coworkers (2018) proposed a “top-down” methodology using an “assembly–fission” reaction under alkaline conditions to covert sublimed sulfur into SQDs, which attained a quantum yield of up to 3.8%.^6^ Moreover, hydrothermal reaction with H_2_O_2_ contributed to improved physicochemical properties of SQDs.^1,12^

Sulfur nanomaterials are used in a wide range of applications including electrocatalysis,^13^ antibacterial agents^14^ and lithium batteries.^15^ To date, there are only a few reports concerning the utilization of SQDs as a fluorescence sensor. For example, water-dispersible SQDs were used for selective Ag^+^ detection with limit of detection of 810 nM,^16^ and as a fluorescent “switch” for detection of Co^2+^ and norfloxacin.^17^ Further, Qiao *et al.* (2020) employed novel sulfur nanodots for Hg^2+^ sensing and *in vitro* cellular tracking.^18^ These reports focused on the utilization of SQDs based on aggregation-caused quenching (ACQ) mechanisms. To our knowledge, research has not explored the utilization of SQDs based on inner-filter effect (IFE) mechanisms. The IFE phenomenon is triggered *via* absorption of excitation and/or emission wavelengths by the absorber in the system. The basic requirement for a highly efficient IFE is good overlap between the absorption band of the absorber and the excitation band (the primary IFE) and/or emission band (the secondary IFE) of the fluorophore.^19^ In fact, IFE is often a source of error in fluorimetry methods. As compared to classical probe strategies, such as intramolecular charge transfer (ICT), photo-induced electron transfer (PET) and fluorescence resonance energy transfer (FRET),^20^ IFE improves sensitivity due to the exponential transformation of probe absorbance into fluorescence intensity changes.^21^ For example, dual-signaling of g-C_3_N_4_ and gold nanoparticles were utilized to detect organophosphorus pesticides in fruit juice based on IFE.^22^ Building upon this previous research, we herein developed a SQD-based “ON–OFF–ON” fluorescence platform for sequential detection of Cr(vi) and ascorbic acid (AA) in environmental and human samples, as well as for bioimaging in embryonic–larval zebrafish (*Danio rerio*) and HeLa cells.

Chromium mainly exists in two stable oxidation states of trivalent chromium (Cr(iii)) and hexavalent chromium (Cr(vi)).^23^ In the industrial processes of tanning, printing/dyeing, smelting and electroplating, a large amount of chromium is released in wastewater.^24^ Specifically, Cr(vi) has high carcinogenic, teratogenic and mutagenic properties, even at low concentrations.^21^ Several analytical methodologies are available for trace detection of Cr(vi), such as spectrophotometry,^25^ chromatography^26^ and atomic absorption spectroscopy.^25^ However, the above methods have limited efficacy for remote/outdoor use due to the requirements of expensive equipment and complicated sample pretreatment procedures.^27^ Therefore, the establishment of a simple, sensitive and selective method for trace Cr(vi) detection is of great significance for routine environmental and biological monitoring.

Ascorbic acid (AA), also known as “vitamin C”, is an organic compound with antioxidant properties.^28^ AA participates in many biosynthetic processes and the reduction of oxidative stress *via* its ascorbic acid oxidase (AAO) substrate.^29^ However, abnormal level (deficiency & excess) may lead to a variety of diseases, such as scurvy, immune system disorders and kidney stones.^30,31^ Common methods for determination of AA include injection spectrophotometry,^32^ electrochemical methods,^33^ liquid chromatography^34^ and chemiluminescence. In contrast to conventional methods, fluorescent nanosensors provide a powerful detection platform for AA detection due to their high sensitivity, simple device requirements and rapid/convenient operation. Although some graphene quantum dots (GQDs) and carbon quantum dots (CQDs) have been developed as nanofluoroprobes for AA, there are no reports using SQDs for detection of AA or the sequential detection of Cr(vi) and AA.

**Scheme 1:**
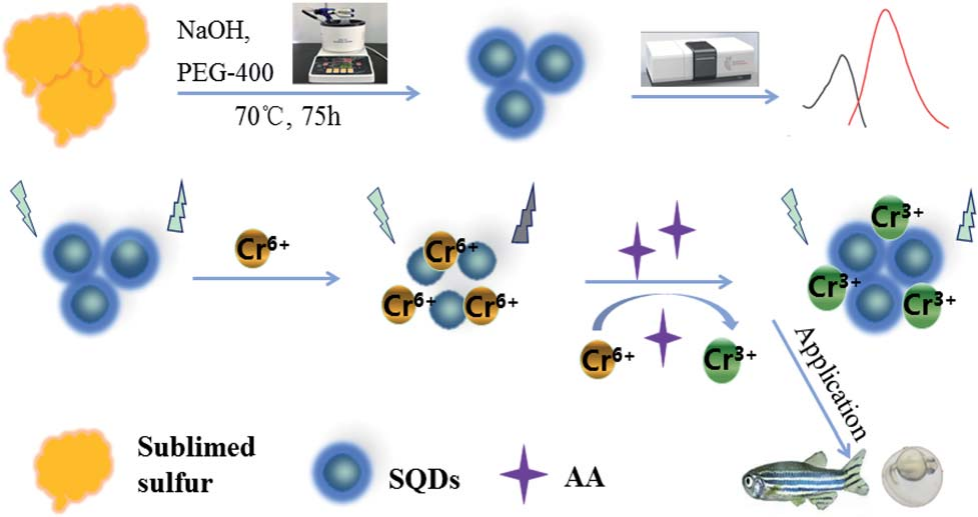
Schematic illustration of the SQDs-based “ON–OFF–ON” fluorescence sensor.

In this investigation, we used an “assembling–fission” top-down method to synthesize highly efficient SQDs. Due to the IFE phenomenon, Cr(vi) has a quenching effect on the fluorescence intensities (FIs) of SQDs, which recovers following redox reaction with AA (Scheme 1). Based on these reaction pathways, SQDs were used as a nanofluoroprobe to sequentially detect Cr(vi) and AA. The as-constructed nanosensor was then employed for bioimaging of both Cr(vi) and AA in living cells and organism using HeLa cell lines and zebrafish embryos/larvae. As a preferred model organism, zebrafish (*Danio rerio*) have the distinct advantages of short growth period, transparent embryos and ease of collection and husbandry.^35^ Bioimaging is a critical component of biological and medical research providing *in situ* observation of the morphological characteristics and abnormal state of cells or specific tissues of organisms. In recent years, bioimaging has been widely applied due to advances leading to high sensitivity, high resolution and fast imaging speed, as well as development of enhanced imaging technologies.^36^

## 2. Experimental section

### 2.1. Preparation of SQDs

SQDs were prepared based on an “assembling–fission” methodology.^6^ In brief, sublimed sulfur powder (1.4 g) and concentrated nitric acid (50 mL) were added to a round-bottomed flask and stirred at 70 °C for 24 h. The mixture was centrifuged at 5000 rpm for 5 min to isolate the sediment, which was washed three times with ultrapure water. Then, 50 mL of ultrapure water, 3 mL of PEG-400 and 4 g of NaOH were added to the flask containing the rinsed precipitate, and stirred at 70 °C for 75 h. The resulting orange-yellow colloids were stored at 4 °C in the dark for further use. When used for bioimaging experiments, the aforementioned SQDs were further dialyzed for 8 h using dialysis membrane (MWCO, 1000 Da) and diluted by two-fold using ultrapure water.

### 2.2. Fluorescence determination of Cr(vi) and AA

For detection of Cr(vi), an aliquot (100 μL) of the above-prepared SQDs was dispersed in 800 μL PBS (pH = 7.0). Then, 100 μL of Cr(vi) (concentration range = 0.005–3.0 mM) were added into the mixture and incubated at ambient conditions for 1 min. Fluorescence spectra were recorded at excitation/emission wavelengths of 395/500 nm.

For detection of AA, an aliquot (100 μL) of Cr(vi) solution (25 mM) was fortified into the mixture containing 100 μL of SQDs and 700 μL PBS (pH 7), and incubated for 1 min to form the SQD/Cr(vi) solution. Then, 100 μL of AA (concentration range = 0.01–7.0 mM) were spiked into the SQD/Cr(vi) mixture, and incubated at room temperature for 15 min. Fluorescence spectra were recorded at excitation/emission wavelengths of 395/500 nm.

### 2.3. Sequential detection of Cr(vi) and AA in real-world samples

Tap water was collected in our laboratory at Suzhou University of Science and Technology and river water from Taihu Lake at Suzhou. Water samples were sequentially filtered through a PVDF filter and 0.45 μm PES membrane filter. Recovery experiments were conducted by fortifying with Cr(vi) concentrations of 0.5, 1.0 and 2.0 mM. Vitamin C tablets were ground into a powder, and an aliquot (100 mg) dissolved into 30 mL of ultrapure water. The analytical performance of the as-constructed “ON–OFF–ON” sensor for determining AA concentrations in human serum and vitamin C tablets was assessed along with recovery from three fortification levels (0.05, 0.5 and 1.5 mM).

### 2.4. Cell culture and cytotoxicity of SQDs

HeLa cells (human cervical cancer cell lines) were cultured in Dulbecco's Modified Eagle's Medium (DMEM), which was composed of 10% FBS and antibiotics (50 units per mL penicillin and 50 units per mL streptomycin). All cells were cultured at 37 °C in a humid atmosphere containing 5% CO_2_. In short, cells were transferred into 96-well plates at a density of 1 × 10^4^ cells per well and incubated for 24 h. Different volumes (5–50 μL) of the pretreated SQDs (described in Section 2.3) were added to each well, and made up to a final constant volume of 200 μL. After 72 h of cell growth, the resulting samples were used for evaluating the cytotoxicity by MTT assay.^37^ Briefly, 20 μL of MTT stock solution (5 mg mL^−1^) in PBS was added to each well. After cellular incubation for 4 h, the medium containing unreacted dye was carefully removed, and 200 μL of DMSO was added to each well to dissolve the blue formazan crystals. The absorbance was measured at 490 nm in a BioTek® Synergy H4 hybrid reader, the absorbance being proportional to the number of living cells in the culture. Cell viability was computed as:
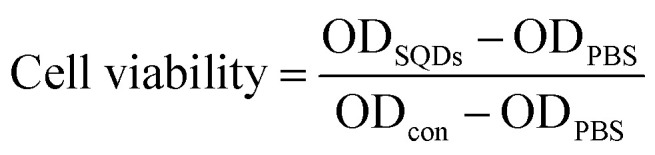
where OD_SQDs_, OD_PBS_ and OD_con_ denote the optical densities in the SQDs, PBS and control solutions, respectively.

### 2.5. Bioimaging of HeLa cells

HeLa cells were seeded into a 12-well plate at a density of 2 × 10^5^ cells per well and cultured for 24 h. Then, the DMEM medium was carefully removed using a pipette, and the sedimented cells were washed 3 times with ice-cold PBS. After washing, HeLa cells were divided into three groups. The first group was incubated with 100 μL of SQD solution for 2 h. The second group was incubated for 15 min in AAO solution (2 U mL^−1^), then 100 μL of SQDs added followed by a 2 h incubation, and finally 100 μL of 25 mM Cr(vi) solution was added followed by a 15 min incubation. In the third group, the cells were incubated with 100 μL of SQDs for 2 h, and then 100 μL of 25 mM Cr(vi) added followed by a 15 min incubation. The cells were fixed with 4% paraformaldehyde at room temperature for 30 min, and then washed 3 times with ice-cold PBS. The resultant cell solution was coated on a slide and dried for observation with a confocal laser microscope.

### 2.6. Bioimaging of zebrafish embryos and larvae

All animal procedures were performed in accordance with the Guidelines for Care and Use of Laboratory Animals of Suzhou University of Science and Technology, Suzhou, China and experiments were approved by the Animal Ethics Committee of Suzhou University of Science and Technology. The embryos (48 hours post fertilization, hpf) and larvae (120 hpf) of wild-type (AB strain) zebrafish were employed in this investigation. Zebrafish were incubated at 28 °C with PTU (1 mM) added to inhibit melanin production for the sake of maintaining transparency of the whole body. Embryos and larvae were allocated to 10 wells in 12-well plates for bioimaging of three experimental groups. In the first group, embryos and larvae were incubated for 2 h in 100 μL of SQDs. In the second group, embryos and larvae were firstly incubated in AAO (2 U mL^−1^) for 15 min, then addition of 100 μL SQDs for another 2 h incubation, and finally fortified with 100 μL of 25 mM Cr(vi) solution for 15 min incubation. In the third group, embryos and larvae were incubated in 100 μL SQDs for 2 h, and then 100 μL of 25 μM Cr(vi) solution was added followed by a 15 min incubation. Following treatment, larvae were anaesthetized with 0.4% MS-222 for 5 min and washed 3 times with PBS. The resulting embryos or larvae were placed on a slide for observation with a laser confocal microscope.

## 3. Results and discussion

### 3.1. Characterization of SQDs

**Fig. 1:**
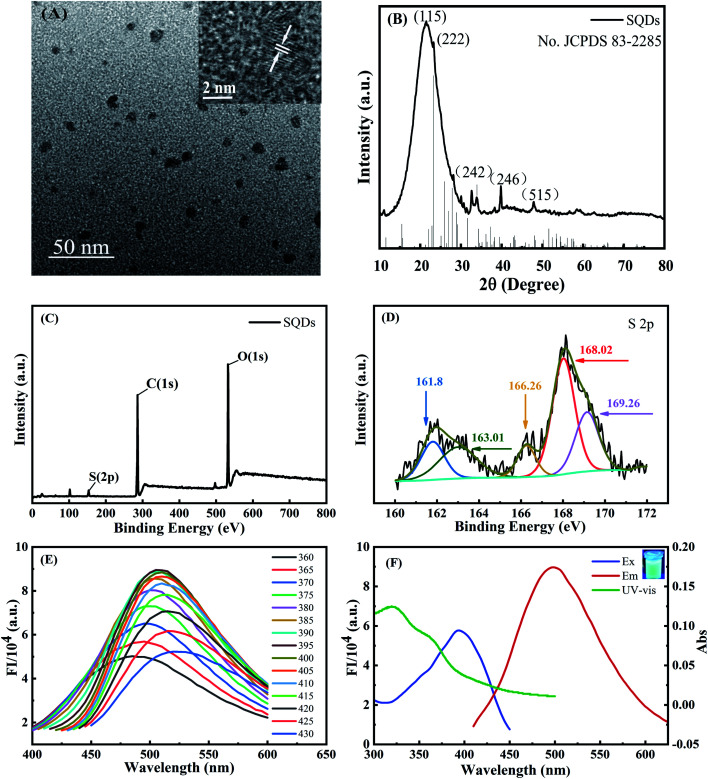
(A) TEM (inset HRTEM); (B) XRD; (C) XPS; (D) S 2p XPS of SQDs; (E) fluorescence spectra at different excitation wavelengths of SQDs; (F) UV-Vis absorption (green line), fluorescent excitation (blue line) and emission spectra (red line); (inset) SQDs irradiated by UV light at 365 nm.

TEM observation demonstrated that the SQDs were homogeneously dispersed in the aqueous solution with a size range of ∼5–10 nm (Fig. 1A). As shown in the inset of Fig. 1A, the interplanar spacing is 0.21 nm, which belongs to the (337) diffraction plane of face-centered S.^18,38^ XRD peaks at 21.3° (115), 23.2° (222), 33.8° (242), 39.5° (246) and 48.1° (515) were attributed to S_8_, which was in general agreement with standard card no. JCPDS 83-2285 (Fig. 1B). These characteristic peaks indicated that a sulfur polycrystalline phase was formed.^18^ XPS characterization of the elemental composition of SQDs indicated peaks were ascribed to S, C and O, respectively (Fig. 1C). The high resolution S 2p at 161.8 eV and 163.01 eV were assigned to atomic sulphur, while the binding energies at 166.26 eV, 168.02 eV and 169.26 eV were attributed to SO^2−^ (2p_2/3_), SO_2_^2−^ (2p_1/2_) or SO_3_^2−^ (2p_2/3_) and SO_3_^2−^ (2p_1/2_), respectively (Fig. 1D).^39,40^ These results provided compelling evidence that the SQDs were composed of atomic sulphur with abundant sulfonyl or sulfonate groups on the surface. FT-IR spectra characterizing surface functional groups revealed a 1650 cm^−1^ peak attributed to the stretching vibration of the C=O group, and along with O–H peaks at 3435 and 1297 cm^−1^ indicate the presence of –COOH (Fig. S1†). The simultaneous existence of SO_2_^2−^, SO_3_^2−^, –COOH and –OH illustrates in part why SQDs possess good water dispersibility.^12^ Additionally, the 2914 cm^−1^ peak reflects the saturation stretching vibration peak of C–H, and the 1456 cm^−1^ and 1352 cm^−1^ peaks are deformation vibration peaks of C–H resulting from the addition of PEG.^6^ Furthermore, the bending vibration of the S–O bond at 829 cm^−1^ confirms the successful synthesis of SQDs.

The fluorescent emission spectra of SQDs were strongly dependent on the excitation wavelengths over the range of 360–430 nm, which arises from the uneven size distribution of quantum size and emission at different excitation displacement (Fig. 1E).^6^ The FIs increased gradually with increasing excitation wavelengths from 360 to 395 nm, followed by a prominent decrease with further increasing wavelengths from 395 to 430 nm. As a result, we chose 395 nm as the optimized excitation wavelength with the maximum emission wavelength of 500 nm (Fig. 1F). For UV-Vis absorption spectra of SQDs (Fig. 1F), two absorption peaks located at 319 and 361 nm were assigned to n → π*, indicating the existence of S_2_^2−^ and S_8_^2−^ species in SQDs.^18^ The FIs of SQDs increased only slightly from pH 5.8 to 8.0 (Fig. S2A†), indicating the relative acid–base stability of these nanodots.^18^ Combining pH optimization results from SQD/Cr(vi) and SQD/Cr(vi)/AA mixed systems, we chose pH 7 for subsequent experiments. Notably, the FI of SQDs at 25 d remained as high as 89.7% relative to its initial values (Fig. S2B†), demonstrating the excellent temporal stability of this nanomaterial. The high stability of SQDs results in part from the addition of PEG-400 as a passivator to stabilize SQDs in their colloidal state.^6^

### 3.2. Fluorescence determination of Cr(vi)

**Fig. 2:**
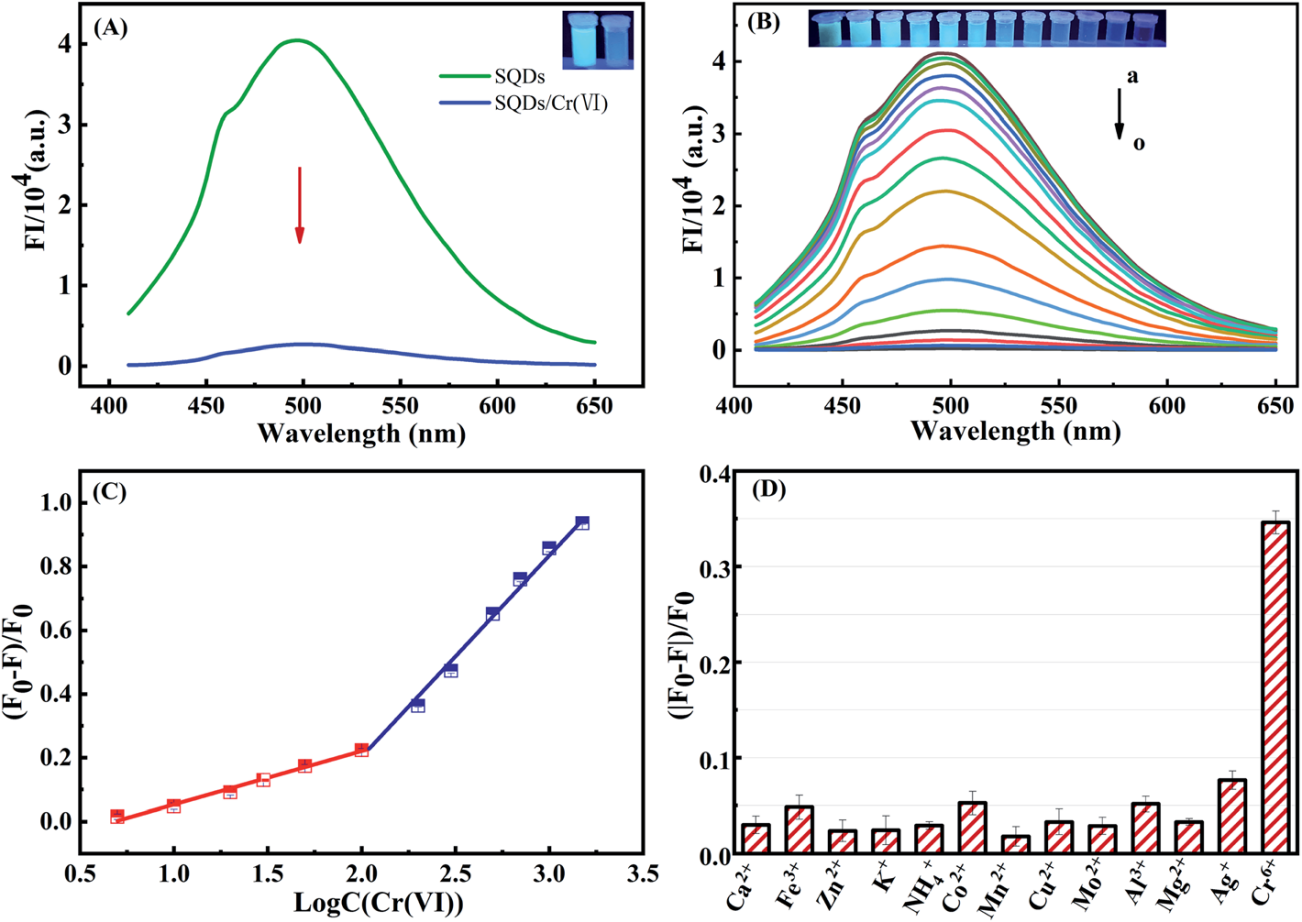
(A) Fluorescence spectra of SQDs and SQDs/Cr(vi). (B) The fluorescence emission spectra of SQDs/Cr(vi) system with varying Cr(vi) concentrations (a → o = 5, 10, 20, 30, 50 (μM) and 0.1, 0.2, 0.3, 0.5, 0.7, 1, 1.5, 2.5, 3 (mM)). (C) Linear relationship between fluorescence emission intensity of SQDs/Cr(vi) and Cr(vi) concentration. (D) Fluorescence response of SQDs for Cr(vi) (0.2 mM) and potential interfering metal ions (1 mM).

The FIs of SQDs were sharply decreased from 4.03 × 10^4^ to 2.67 × 10^3^ a.u. upon addition of Cr(vi), indicating that Cr(vi) significantly quenches the fluorescence of SQDs (Fig. 2A). Therefore, we posit the potential for SQDs as a fluorescence probe to detect Cr(vi) based on the “ON–OFF” principle. To obtain high performance of the fluorescent assay for Cr(vi) detection, several key variables were sequentially optimized. Upon addition of Cr(vi), the fluorescence quenching efficiency increased up to 98.2% after a 1 min incubation time, and then remained nearly unchanged (Fig. S3A†). Consequently, we selected 1 min as the optimized incubation time for the SQDs/Cr(vi) system. When solution pH increased from 5.8 to 6.4, the quenching efficiency of Cr(vi) increased gradually from 97.8 to 98.3%, and subsequently remained nearly stable as pH was further increased from 6.4 to 8.0 (Fig. S3B†). Based on these optimization trials, we chose pH 7 for subsequent analyses using the SQDs/Cr(vi) system.

Under optimized conditions (pH = 7 and 1 min incubation time), the analytical performance of the “ON–OFF” fluorescent assay was evaluated in terms of coefficient of determination (*R*^2^), linear range (LR) and limits of detection (LOD). The fluorescence of SQDs in the presence of Cr(vi) is shown in Fig. 2B. The FIs of SQDs gradually decreased with increasing Cr(vi) concentrations from 5 to 3000 μM (Fig. 2B), exhibiting a good linear relationship from 5 to 100 μM and 200 to 1500 μM with *R*^2^ of 0.9961 and 0.9922, respectively (Fig. 2C). The two stage linear ranges of this detection method was similar to some literatures.^31,42^ The LOD was 1.5 μM, computed as 3 times the standard deviation of the FI of the blank (*n* = 12) divided by *K*_SV_ (Stern–Volmer constant).^37^ This value compares to the World Health Organization (WHO) drinking water standard for Cr(vi) of <50 μg L^−1^ (<0.96 μM).^20^ The LR of the “ON–OFF” system spanned a wide range of 5–1500 μM for Cr(vi) detection (Table S1†). Thus, it is concluded that the as-constructed nanofluoroprobe can satisfy the technical requirements for trace-level detection of Cr(vi) in drinking water, showing great potential for low-cost, routine monitoring of Cr(vi) in environmental waters. As compared to other probes, the SQD-based probe provided a wider LR and similar LOD for Cr(vi) detection (Table S1†).

To assess potential matrix interferences for the fluorescent assay, the effects of common major cations (Ca^2+^, Fe^3+^, Zn^2+^, K^+^, NH_4_^+^, Co^2+^, Mn^2+^, Cu^2+^, Mo^2+^, Al^3+^, Mg^2+^ and Ag^+^) on the FIs of SQDs were evaluated. The fluorescence quenching efficiency was ∼35% in the presence of 0.2 mM Cr(vi), while they were less than 7.0% in the presence of the other cations (at 1.0 mM), thereby being 5-fold lower than that of Cr(vi) (Fig. 2D). Consequently, the fluorescent assay based on SQDs showed excellent selectivity for determination of Cr(vi) with minimal matrix interference from common cations.

### 3.3. Quenching mechanisms of SQDs by Cr(vi)

**Fig. 3:**
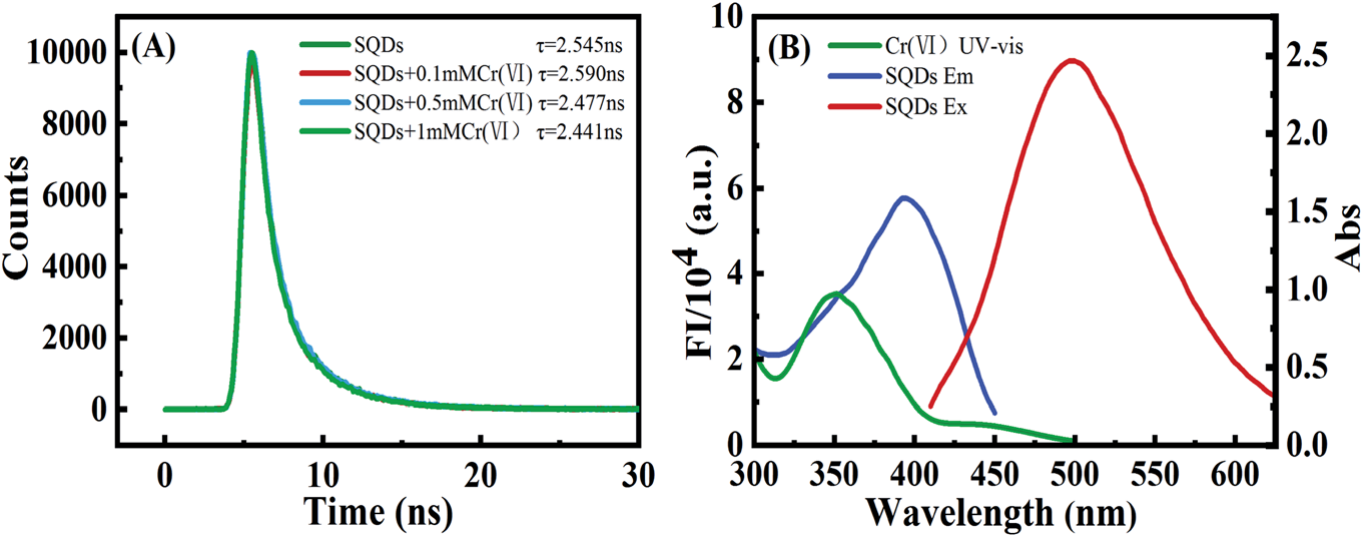
(A) Fluorescence lifetimes of SQDs with varying concentrations of Cr(vi). (B) UV-Vis absorption (green line) of 0.1 mM Cr(vi), fluorescent excitation (blue line) and emission spectra (red line) of SQDs in aqueous solution.

To explore the quenching mechanism, fluorescence lifetimes (FLs) of SQDs were tested at contrasting Cr(vi) concentrations. Fluorescence decay curves were well fit by a double-exponential function (eqn (1)) (Fig. 3A):1
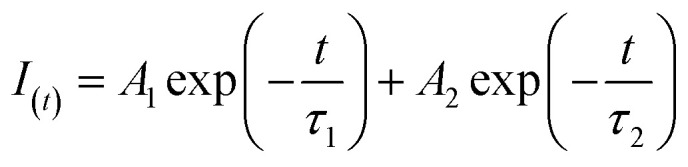
where *τ*_1_ and *τ*_2_ are the time constants of the two radiative decay channels; and *A*_1_ and *A*_2_ are the corresponding amplitudes. The calculated FLs of SQDs remained nearly constant at 2.545 ns (2.441–2.590 ns). The slight changes in FLs indicate a static quenching between SQDs and Cr(vi).^41,42^ This phenomenon excludes the FRET process between SQDs and Cr(vi) because the FLs of the mixed system would be substantially changed by an efficient FRET process.^22^ Additionally, the UV-Vis absorption spectra of Cr(vi) partially overlapped with the fluorescence excitation wavelength of SQDs (Fig. 3B), suggesting that the quenching of SQDs by Cr(vi) was not due to charge transfer.^43,44^ Therefore, we conclude that the FIs of SQDs were quenched by Cr(vi) through IFE mechanisms. In order to further verify the interaction between SQDs and Cr(vi), the UV-Vis absorption spectra and zeta potential test were conducted. As compared to the UV-Vis absorption spectra of SQDs, there was an obvious red shift upon addition of Cr(vi) (Fig. S4A†), indicating the formation SQDs/Cr(vi) complexes due to coordination of Cr(vi) with O-containing groups of SQDs. As depicted in Fig. S4B,† the zeta potential of SQDs increased from −10.45 to −3.5 mV upon addition of 2.5 mM Cr(vi), demonstrating the electrostatic interactions between SQDs and Cr(vi). Based on the above findings, the electrostatic and coordination interactions between SQDs and Cr(vi) led to the quenched fluorescence of CuNCs by IFE mechanisms.^31,45^

### 3.4. Fluorescence determination of AA by the “ON–OFF–ON” mixed system

**Fig. 4:**
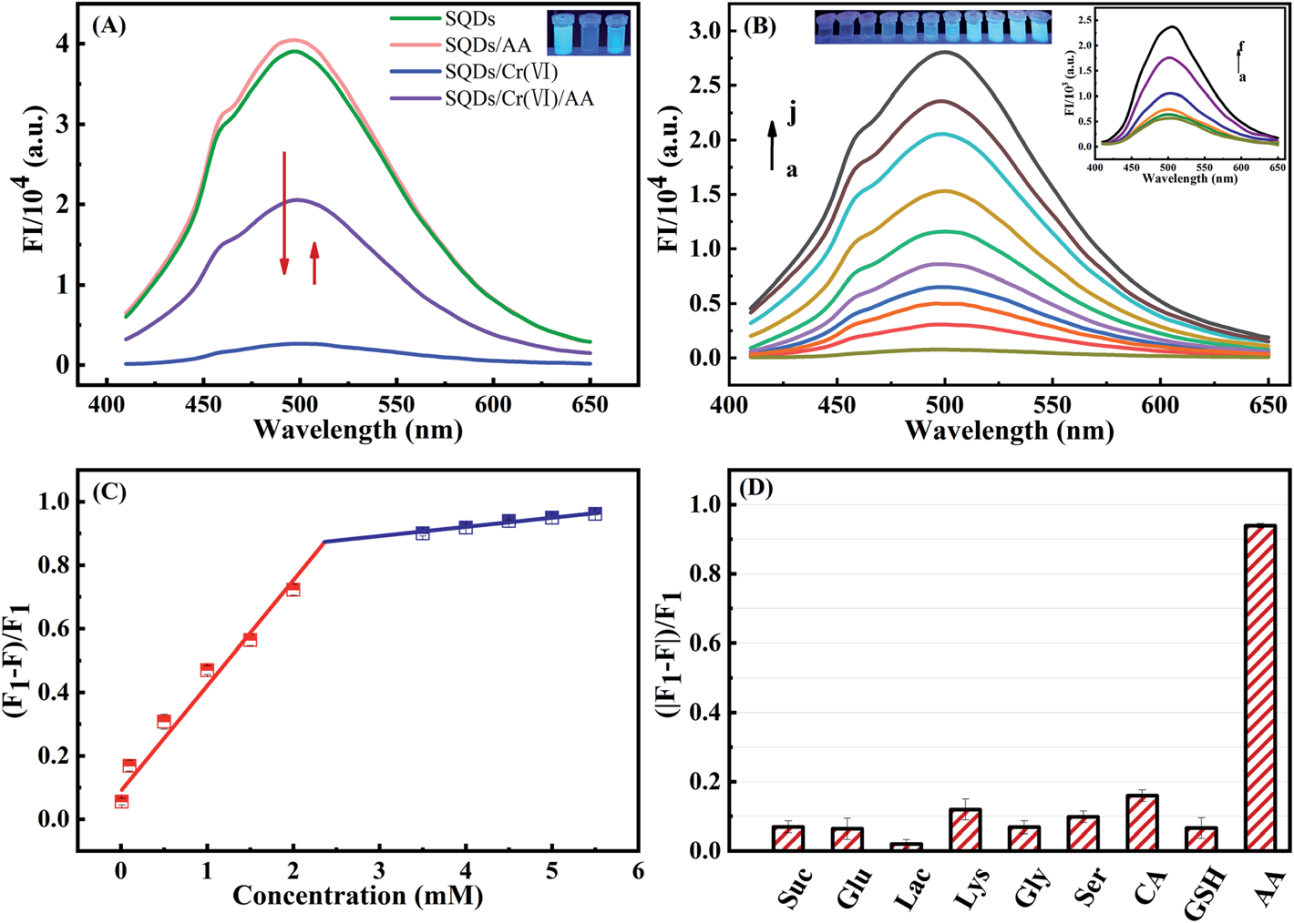
(A) Fluorescence spectra of SQDs, SQDs/AA, SQDs/Cr(vi) and SQDs/Cr(vi)/AA systems. (B) Fluorescence emission spectra of SQDs/Cr(vi)/AA system with varying AA concentrations (a → f = 0.01, 0.1, 0.5, 1, 1.5 and 2 mM (inset graph); and a → j = 0.01, 3, 3.5, 4, 4.5, 5, 5.5, 6, 6.5 and 7 mM). (C) Linear relationship between fluorescence emission intensity of SQDs/Cr(vi)/AA and AA concentration. (D) Fluorescence response of SQDs/Cr(vi) in the presence of AA (5 mM) and potential interfering compounds (10 mM).

The FIs of SQDs were sharply decreased from 4.03 × 10^4^ to 5.63 × 10^2^ a.u. upon addition of Cr(vi). Nevertheless, the introduction of AA (5 mM) significantly recover the FIs to values as high as 2.05 × 10^4^ a.u. owing to the reduction of Cr(vi) to Cr(iii) by AA (Fig. 4A). In sharp contrast, the FIs of SQDs remained nearly unchanged upon addition of AA in the absence of Cr(vi). These results provide strong evidence that the SQDs could be suitable for development of an “ON–OFF–ON” fluorescent platform for detection of AA.

To enhance performance of the as-constructed “ON–OFF–ON” nanofluoroprobe, the operational variables of pH and incubation time were optimized in the SQD/Cr(vi)/AA system. A pH value of 7.0 and a 15 min incubation time provided the best recovery efficiency for the FIs of SQDs and were adopted for subsequent experiments (Fig. S5†). Using the optimized conditions, the analytical performance of the “ON–OFF–ON” fluorescent platform was evaluated. The FIs of the SQDs/Cr(vi) system recovered gradually with increasing AA concentrations from 0.01 to 7 mM (Fig. 4B). A good linear relationship existed between the FIs of the SQDs/Cr(vi) system and AA concentrations from 0.01 to 2 mM and 3 to 5.5 mM with *R*^2^ values of 0.9731 and 0.9662, respectively. The LOD was calculated to be 3 μM, and the LR spanned a wide range of 10–5500 μM (Table S2†). The as-constructed “ON–OFF–ON” system provided a wider linear detection range and comparable LOD for AA quantification compared to other methods (Table S2†).

To assess potential matrix interferences for the as-constructed “ON–OFF–ON” fluorescent platform for AA detection, some common co-existing compounds were appraised: Suc, Glu, Lac, Lys, Gly, Ser, CA and GSH. A 5 mM AA concentration yielded a recovered efficiency of up to 93.9% while all other compounds (10 mM) produced recovery rates <15% (Fig. 4D). These results demonstrate that the “ON–OFF–ON” platform displayed excellent selectivity for AA with little interference potential from common co-existing compounds.

### 3.5. Application to real-world samples

To demonstrate the practical applicability of the as-constructed fluorescent assay for real-world samples, we used the platform to detect Cr(vi) in tap and river waters, and AA concentrations in human serum and vitamin C tablets. As for the detection of Cr(vi), the fortified recovery was in the range of 98.6–105.6% with RSDs <3.7% (Table 1). AA concentrations in unfortified human serum and vitamin C tablets were 84.1 and 166.2 μM, respectively (Table 2). At three fortification levels (50–1500 μM), AA recoveries varied between 97.0% and 102.2% with RSDs <5.3%. These data provided compelling evidence for the efficacy of the newly developed nanofluoroprobe for sequential detection of Cr(vi) and AA with high sensitivity and precision in relevant real-world samples with complex matrices.

**Table 1:** Detection of Cr(vi) in real-world water samples using SQD-based “ON–OFF” fluorescence platform

Sample	Added Cr(vi) (mM)	Found Cr(vi) (mM)	Recovery (%)	Intra-day precision (RSD, %)	Inter-day precision (RSD, %)
River water	0	—	—	—	—
	0.01	0.0106	105.6	3.5	3.7
	0.05	0.0493	98.6	1.9	2.1
	0.10	0.1029	102.9	1.8	2.9
Tap water	0	—	—	—	—
	0.01	0.0104	103.9	2.2	2.6
	0.05	0.0494	98.7	1.3	2.1
	0.10	0.1023	102.3	1.2	2.0

**Table 2:** Detection of AA in human serum and vitamin C tablets using SQDs/Cr(vi)-based “ON–OFF–ON” fluorescence platform

Sample	Added AA (mM)	Found AA (mM)	Recovery (%)	Intra-day precision (RSD, %)	Inter-day precision (RSD, %)
Human serum	0	0.08	—	4.9	5.3
	0.05	1.11	102.9	3.9	4.3
	0.5	0.60	102.2	2.6	3.8
	1.5	1.57	99.3	2.1	3.3
Vitamin C tablets	0	0.17	—	3.3	4.1
	0.05	0.22	101.9	2.0	2.7
	0.5	0.65	97.0	2.9	2.6
	1.5	1.68	101.3	1.0	2.2

### 3.6. Bioimaging of SQDs in HeLa cells and zebrafish embryos/larvae

**Fig. 5:**
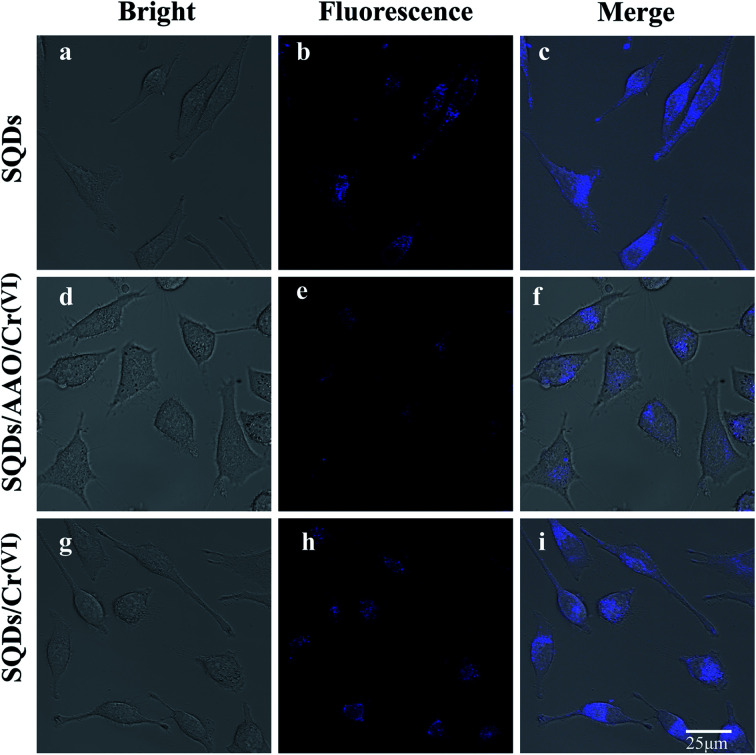
Fluorescence images of live HeLa cells observed by laser confocal microscope. Notes: (1) fluorescence images (a–c) of HeLa cells incubated with SQDs. (2) Fluorescence images (d–f) of HeLa cells incubated with AAO (2 U mL^−1^) in the SQDs/Cr(vi) system. (3) Fluorescence images (g–i) of HeLa cells in SQDs/Cr(vi) system. (4) Different emission channels: bright field (first column); blue light channel (second column, excitation wavelength 345 nm); merged (third column, combined bright field and blue light channel). (5) AAO denotes abbreviation for ascorbic acid oxidase.

To assess the real-world application of SQD-based “ON–OFF–ON” sensors in biological systems, we firstly evaluated the cytotoxicity of SQDs by the MTT colorimetric method. When HeLa cells were incubated for 24 h at different SQD concentrations (0–50 μL of diluted stock solution), the cellular survival rates in all treatment groups were more than 86% (Fig. S6†), highlighting that SQDs exhibit low toxicity and good biocompatibility. Therefore, we posit that SQDs could be utilized for live cell fluorescence imaging. As shown in Fig. S7,† in comparison with SQDs, the blue light channel and combined channels in HeLa cells had very weak fluorescence intensity, indicating the autofluorescence of HeLa cells had little interference to this experiment. The fluorescence imaging of SQDs in HeLa cells was observed using a laser confocal microscope (Fig. 5). Under bright field illumination, no blue fluorescence was observed in any of the three systems (SQDs, SQDs/AAO/Cr(vi) and SQDs/Cr(vi)) (Fig. 5a, d and g). Using the blue light channel, blue fluorescence appeared in all three systems; however, the FIs varied greatly among systems. The strongest FI was observed in the SQDs system (Fig. 5b), followed by the SQDs/Cr(vi) (Fig. 5h) and SQDs/AAO/Cr(vi) systems (Fig. 5e). The introduction of AAO, which exists in cells, in the SQDs/AAO/Cr(vi) system inhibited the reduction action of AA preventing conversion of Cr(vi) to Cr(iii). Thus, the quenched fluorescence of the SQDs/Cr(vi) system was not restored in cells, leading to weak FIs in the SQDs/AAO/Cr(vi) system (Fig. 5e). In comparison, cellular AA reduced Cr(vi) to Cr(iii) resulting in improved FIs in the SQDs/Cr(vi) system (Fig. 5h). Under merged channels, similar changes in FI trends occurred in the three systems, as found with the blue light channel (Fig. 5 – third column). However in the same system, the merged channel produced weaker FIs than the blue light channel due to the existence of the bright field (Fig. 5c, f and i). These examples illustrate the efficacy of the SQD-based fluorescent sensor for a range of cell imaging applications.

**Fig. 6:**
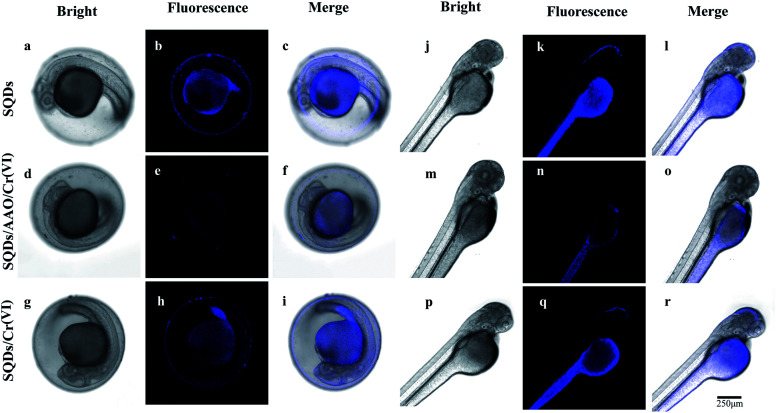
(1) Fluorescence images (a–c and j–l) of zebrafish embryos and larvae incubated with SQDs. (2) Fluorescence images (d–f and m–o) of zebrafish embryos and larvae incubated in AAO (2 U mL^−1^) in the SQDs/Cr(vi) system. (3) Fluorescence images (g–i and p–r) of zebrafish embryos and larvae in the SQDs/Cr(vi) system. (4) Different emission channels: bright field (first column); blue light channel (second column, excitation wavelength 345 nm); merged (third column, combined bright field and blue light channel). (5) AAO denotes abbreviation for ascorbic acid oxidase.

In zebrafish embryo and larvae, a blue fluorescence appeared similar to that in the HeLa cells (Fig. 6). Under blue light and merged channels, blue fluorescence occurred in the yolk of embryos (Fig. 6), as well as the yolk and head of larvae (Fig. 6k, i, q and r). In contrast, no blue fluorescence was observed in the larval head using the SQDs/AAO/Cr(vi) system due to no fluorescent recovery (Fig. 6n and o). The grey yolk under the bright field became blue under the blue light channel. The strongest FIs in embryos and larvae were observed in the SQD system (Fig. 6b and k) under the blue light channel, followed by the SQDs/Cr(vi) (Fig. 6h and q) and SQDs/AAO/Cr(vi) systems (Fig. 6e and n). The changing FI trends among the three systems are explained by the same mechanisms described above for the HeLa cells. These bioimaging pictures show that the as-constructed “ON–OFF–ON” nanofluoroprobe can be successfully applied for direct observation of Cr bioaccumulation in model organisms, and thus has prospect for wide application in biomedical fields.

## 4. Conclusions

A simple synthesis method was used to prepare stable SQDs based on the “assembling–fission” mechanism. We used HRTEM, FTIR, XPS, UV-Vis absorption and fluorescent spectroscopy to rigorously characterize the morphology, composition and optical properties of the SQDs. Due to IFE phenomenon, the fluorescence of SQDs was efficiently quenched by Cr(vi) due to formation of SQD/Cr(vi) complexes. The IFE-based quenching mechanism was confirmed by changes in FLs and overlapping phenomenon between Cr(vi) absorption and excitation of SQDs. By utilizing the reduction action of AA to convert Cr(vi) to Cr(iii), a SQD-based “ON–OFF–ON” platform was constructed for sequential detection of Cr(vi) and AA with LODs of 1.5 and 3 μM, respectively, in environmental/potable waters, human serum and vitamin C tablets. Compared to previously reported nanoprobes, the newly developed fluorescent platform displayed a wide linear range, high selectivity for Cr(vi) and AA, and minimal matrix interferences. The efficacy of the nanofluoroprobe was successfully tested for Cr(vi) and AA bioimaging detection in HeLa cell lines and zebrafish embryos/larvae. Notably, a distinct blue fluorescence was displayed in yolk of embryos along with yolk and head of larvae, demonstrating the feasibility for *in situ* observation of Cr(vi) bioaccumulation by bioimaging. Consequently, the SQD-based nanosensor has great prospects for application in water quality monitoring and biomedical fields.

## Conflicts of interest

There are no conflicts to declare.

## Supplementary Material

RA-011-D1RA00401H-s001
