# Supplementary material for: Sulfur quantum dot-based “ON–OFF–ON” fluorescence platform for detection and bioimaging of Cr(vi) and ascorbic acid in complex environmental matrices and biological tissues
Source: RSC Adv. 2021 Mar 11;11(18):10572–81. doi: 10.1039/d1ra00401h (PMC8695692; doi:10.1039/d1ra00401h)
Supplement: RA-011-D1RA00401H-s001 [file RA-011-D1RA00401H-s001.pdf]

Electronic Supplementary Material (ESI) for RSC Advances.
This journal is © The Royal Society of Chemistry 2021

Sulfur quantum dot-based "ON-OFF-ON" fluorescence platform for detection and bioimaging of Cr(VI) and ascorbic acid in complex environmental matrices and biological tissues

Mengke Xia[1], He Mei[2], Qiuhui Qian[1], Randy A. Dahlgren[2], Ming Gao[1]*, Xuedong Wang[1#]

[1] School of Environmental Science and Engineering, Suzhou University of Science and Technology, Suzhou 215009, China

[2] College of Public Health and Management, Wenzhou Medical University, Wenzhou 325035, China

[#]Corresponding author: Xuedong Wang, Email:zjuwxd@163.com, Phone: +86-5126809 5950. Fax: +86-512-6809 5950

*Co-coresponding author: Ming Gao, Email:gaoming@usts.edu.cn

Submitted to *RSC Advances*

March 05, 2021

Table S1. Comparison of SQDs-based "ON-OFF" method with previously reported methods for Cr(VI) detection

Methods	Linear range (μM)	Detection limit (μM)	Ref.
CD fluorescence sensor	2-180	2.10	[1]
Dual emissive carbon dots	2-300	0.40	[2]
Cobalt(II)-doped carbon dots	5-125	1.17	[3]
GQD-modified membranes	1-500	0.19	[4]
Au-Ag-Pt three material electrodes	2-200	0.90	[5]
SQD-based "ON-OFF"	5-1500	1.50	This work

Table S2. Comparison of SQDs-based "ON-OFF" method with previously reported methods for AA detection

Methods	Linear range (μM)	Detection limit (μM)	Ref.
CdTe quantum dots	10-250	1.30	[6]
AuNCs-PbS quantum dots	3.0-40	1.50	[7]
P, N-CDs quantum dots	5.0-200	1.35	[8]
N, S co-doped carbon quantum dots/Fe^{3+}	10-200	4.96	[9]
LaF_3:Ce, Tb nanoparticles	8-100	2.40	[10]
Carbon dot nanosensor	30-100	1.20	[11]
SQD/Cr(VI)-based "ON-OFF-ON"	10-5500	3.00	This work

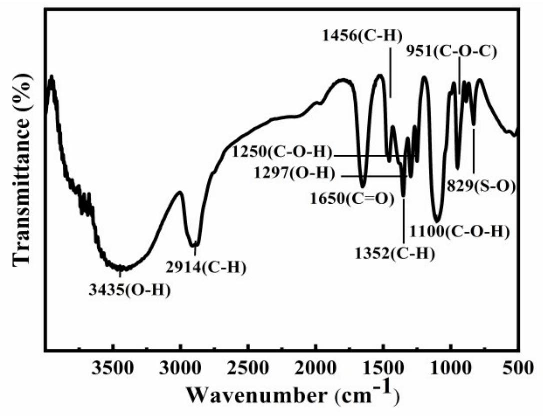

Fig. S1. FT-IR of SQDs

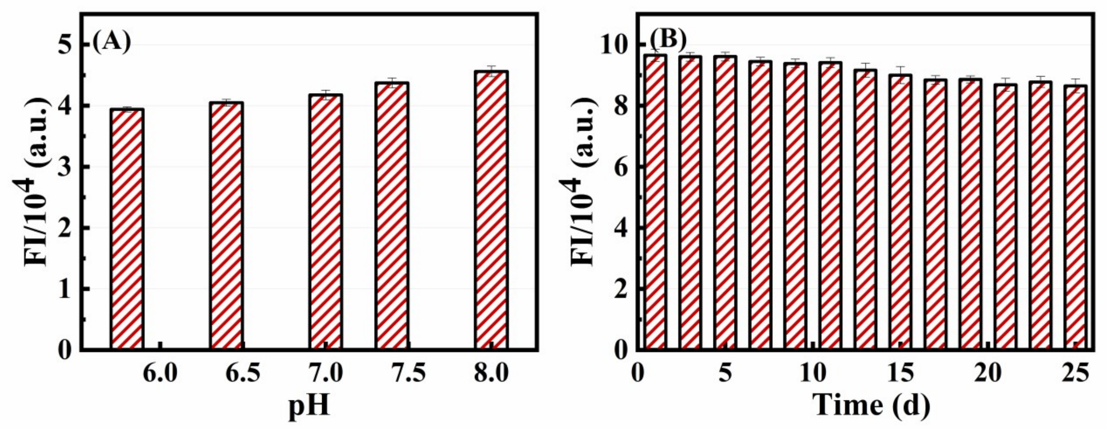

Fig. S2. (A) Effects of pH on the FIs of SQDs. (B) FI stability of SQDs tested

Note: (1) FI indicates abbreviation for fluorescence intensity. (2) All values were reported as mean ±std (n = 3).

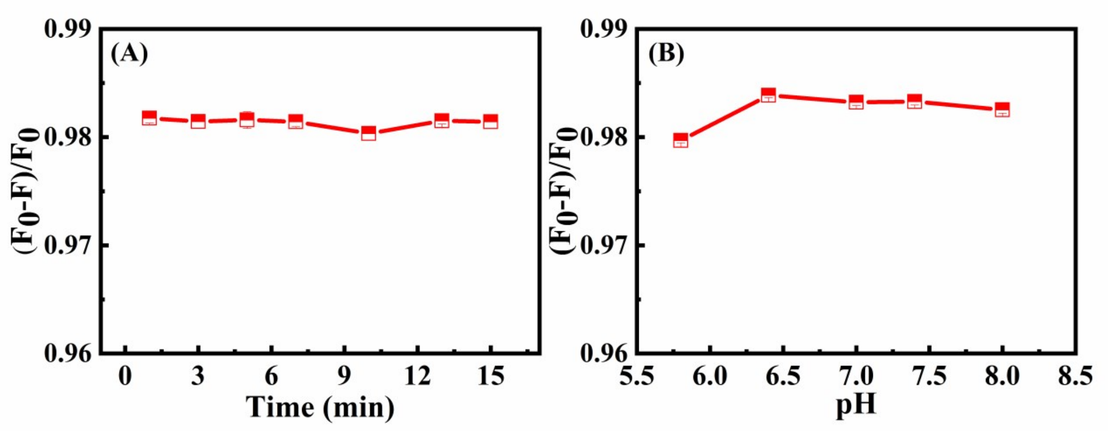

Fig. S3. (A) Effects of reaction time on the FIs of the SQDs/Cr(VI) system. (B) Effects of pH on the FIs of SQDs and SQDs/Cr(VI) systems

Note: (1) Cr(VI) concentration was 25 mM in the SQDs/Cr(VI) system. (2) All values were reported as mean ±std (n = 4).

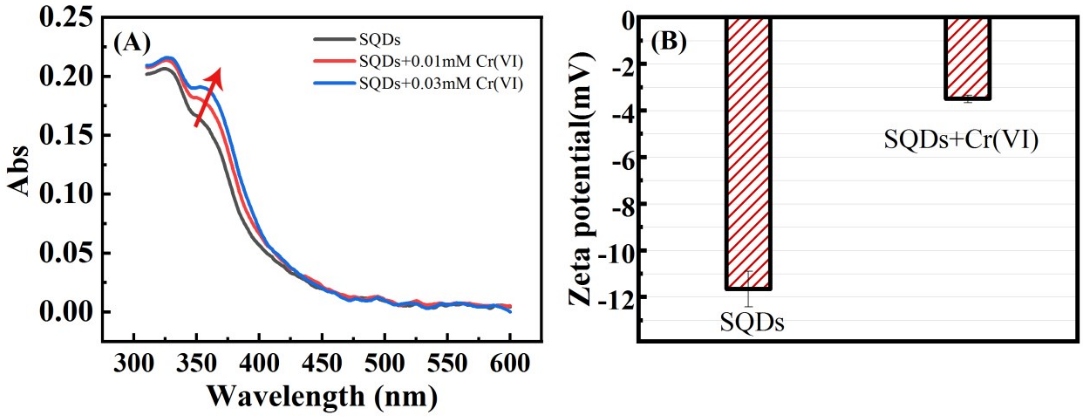

Fig. S4. (A) UV-Vis spectrum of SQDs in the presence of different concentrations Cr(VI); (B) Zeta potential of SQDs and SQDs/Cr(VI) **Note:** Cr(VI) concentrations was 25 mM

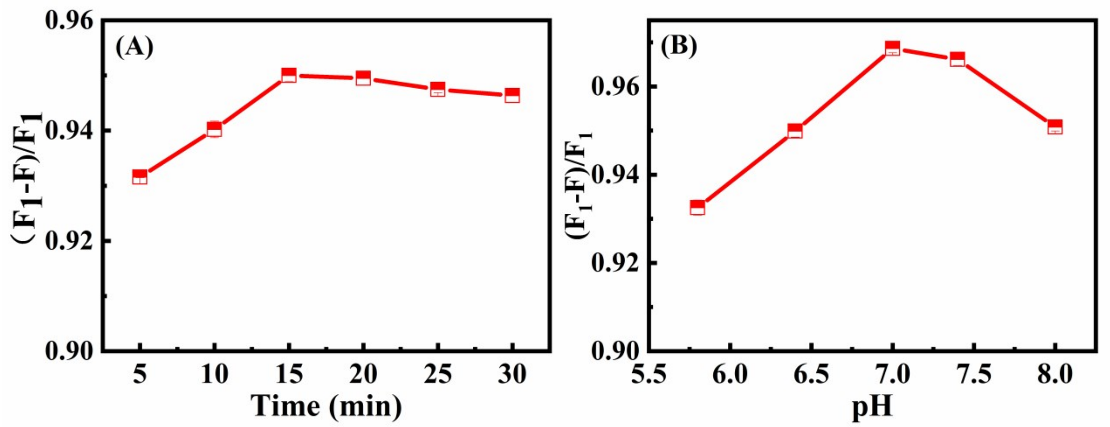

Fig. S5. (A) Effects of reaction time on the FIs of the SQDs/Cr(VI)/AA system. (B) Effects of pH on the FIs of the SQDs and SQDs/Cr(VI)/AA systems

Note: (1) Cr(VI) and AA concentrations were 25 and 50 mM, respectively. (2) All values were reported as mean ±std (n = 4).

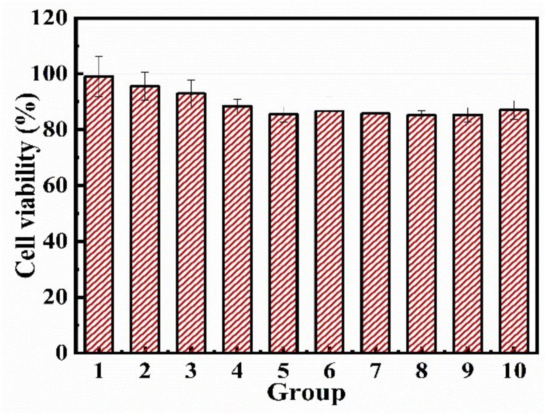

Fig. S6. Effects of the SQDs amount on HeLa cell viability

Notes: (1) Incubation time of HeLa cells was 24 h after dialysis with fortified volumes of 0, 5, 10, 20, 24, 28, 32, 36, 40 and 50 μL for groups 1-10, respectively, in a total constant volume of 200 μL. (2) All values were reported as mean ±std (n = 8).

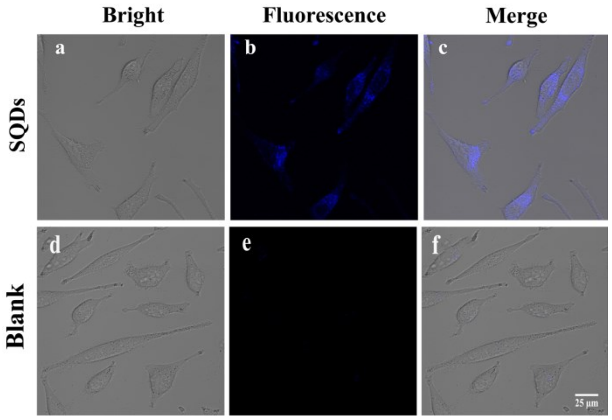

Fig. S7. Fluorescence imaging of HeLa cell with (a-c) and without SQDs (d-f)

References

1. C. Li, W. Liu, X. Sun, W. Pan and J. Wang, Sens. Actuators B: Chem., 2017, 252, 544-553.
2. Y. Ma, Y. Chen, J. Liu, Y. Han, S. Ma and X. Chen, Talanta, 2018, 185, 249-257.
3. H. Y. Zhang, Y. Wang, S. Xiao, H. Wang, J. H. Wang and L. Feng, Biosens. Bioelectron., 2017, 87, 46-52.
4. P. M. Carrasco, I. García, L. Yate, R. Tena Zaera, G. Cabañero, H. J. Grande and V. Ruiz, Carbon, 2016, 109, 658-665.
5. D. Li, J. Li, X. Jia, Y. Xia, X. Zhang and E. Wang, Anal. Chim. Acta, 2013, 804, 98-103.
6. J. Zhu, Z.-J. Zhao, J.-J. Li and J.-W. Zhao, J. Lumin., 2017, 192, 47-55.
7. P. Zhao, K. He, Y. Han, Z. Zhang, M. Yu, H. Wang, Y. Huang, Z. Nie and S. Yao, Anal. Chem., 2015, 87, 9998-10005.
8. X. Gong, Y. Liu, Z. Yang, S. Shuang, Z. Zhang and C. Dong, Anal Chim Acta, 2017, 968, 85-96.
9. X. Luo, W. Zhang, Y. Han, X. Chen, L. Zhu, W. Tang, J. Wang, T. Yue and Z. Li, Food Chem., 2018, 258, 214-221.
10. C. Mi, T. Wang, P. Zeng, S. Zhao, N. Wang and S. Xu, Anal. Methods, 2013, 5, 1463.
11. M. Zheng, Z. Xie, D. Qu, D. Li, P. Du, X. Jing and Z. Sun, ACS Appl. Mater. Interfaces, 2013, 5, 13242-13247.
